# Association of lymph-node antigens with lower Gag-specific central-memory and higher Env-specific effector-memory CD8^+^ T-cell frequencies in a macaque AIDS model

**DOI:** 10.1038/srep30153

**Published:** 2016-07-25

**Authors:** Hiroshi Ishii, Saori Matsuoka, Takushi Nomura, Midori Nakamura, Teiichiro Shiino, Yuko Sato, Naoko Iwata-Yoshikawa, Hideki Hasegawa, Kazuta Mizuta, Hiromi Sakawaki, Tomoyuki Miura, Yoshio Koyanagi, Taeko K. Naruse, Akinori Kimura, Tetsuro Matano

**Affiliations:** 1 AIDS Research Center, National Institute of Infectious Diseases, Tokyo 162-8640, Japan; 2 Center for AIDS Research, Kumamoto University, Tokyo 162-8640, Japan; 3 Department of Pathology, National Institute of Infectious Diseases, Tokyo 162-8640, Japan; 4 Institute for Virus Research, Kyoto University, Kyoto 606-8507, Japan; 5 Department of Molecular Pathogenesis, Medical Research Institute, Tokyo Medical and Dental University, Tokyo 113-8510, Japan; 6 The Institute of Medical Science, The University of Tokyo, Tokyo 108-8639, Japan

## Abstract

Virus-specific CD8^+^ T cells exert strong suppressive pressure on human/simian immunodeficiency virus (HIV/SIV) replication. These responses have been intensively examined in peripheral blood mononuclear cells (PBMCs) but not fully analyzed in lymph nodes (LNs), where interaction between CD8^+^ T cells and HIV/SIV-infected cells occurs. Here, we investigated target antigen specificity of CD8^+^ T cells in LNs in a macaque AIDS model. Analysis of virus antigen-specific CD8^+^ T-cell responses in the inguinal LNs obtained from twenty rhesus macaques in the chronic phase of SIV infection showed an inverse correlation between viral loads and frequencies of CD8^+^ T cells with CD28^+^ CD95^+^ central memory phenotype targeting the N-terminal half of SIV core antigen (Gag-N). In contrast, analysis of LNs but not PBMCs revealed a positive correlation between viral loads and frequencies of CD8^+^ T cells with CD28^−^CD95^+^ effector memory phenotype targeting the N-terminal half of SIV envelope (Env-N), soluble antigen. Indeed, LNs with detectable SIV capsid p27 antigen in the germinal center exhibited significantly lower Gag-N-specific CD28^+^ CD95^+^ CD8^+^ T-cell and higher Env-N-specific CD28^−^CD95^+^ CD8^+^ T-cell responses than those without detectable p27. These results imply that core and envelope antigen-specific CD8^+^ T cells show different patterns of interactions with HIV/SIV-infected cells.

CD8^+^ T-cell responses directed against varieties of viral antigens are induced following human immunodeficiency virus (HIV) infection. These CD8^+^ T cells play a central role in the resolution from acute-phase viremia^1^,^2^,^3^,^4^ but mostly permit persistent HIV replication, leading to AIDS progression. High-frequency CD8^+^ T cells observed in the chronic phase of HIV infection are not sufficient for virus control^5^,^6^,^7^, and highly effective CD8^+^ T cells are considered important for prevention of disease progression. Several human leukocyte antigen (HLA) or major histocompatibility complex class I (MHC-I) alleles are known to be associated with effective CD8^+^ T-cell responses and lower viral loads in HIV infection^8^,^9^,^10^,^11^. Furthermore, previous vaccine trials in macaque AIDS models have shown possible reduction in post-challenge viral loads by induction of effective CD8^+^ T-cell responses^12^,^13^,^14^,^15^. These imply that HIV replication may be controlled by potent CD8^+^ T-cell responses^16^.

Individual viral proteins show different kinetics of expression and degradation, which can affect efficiency of protein-derived epitope presentation to CD8^+^ T cells. Thus, some viral proteins may be the targets for effective CD8^+^ T cells frequently, but others not. Analysis of HIV controllers possessing protective HLA class I alleles such as HLA-B*27 and HLA-B*57 found potent CD8^+^ T-cell responses targeting HIV core Gag epitopes (such as HLA-B*27-restricted Gag_263–272_ KK10 and HLA-B*57-restricted Gag_240–249_ TW10) contributing to HIV control^17^,^18^,^19^. Studies in macaque AIDS models have indicated simian immunodeficiency virus (SIV) control by Gag antigen-specific CD8^+^ T-cell responses^12^,^20^,^21^. In addition to these studies on HIV/SIV controllers, analysis in a large cohort of HIV-infected individuals showed that Gag-specific CD8^+^ T-cell responses are associated with lower viral loads^22^,^23^,^24^.

Understanding of the target protein profiles of effective CD8^+^ T cells are important in the development of an intervention strategy toward HIV-1 control. Virus-specific CD8^+^ T-cell responses have been examined intensively in peripheral blood but not fully analyzed in lymph nodes (LNs) where interaction between CD8^+^ T cells and virus-infected cells occurs, although several studies investigated those in LNs (refs 25 and 26). In the present study, we investigated target antigen profiles of LN-derived CD8^+^ T cells in the chronic phase of SIV infection in rhesus macaques. Our analysis indicated that viral loads were correlated inversely with SIV core Gag antigen-specific central-memory but positively with SIV envelope (Env) antigen-specific effector-memory CD8^+^ T-cell frequencies in LNs.

## Results

### CD8^+^ T-cell responses targeting individual SIV antigens in LNs

**Figure 1:**
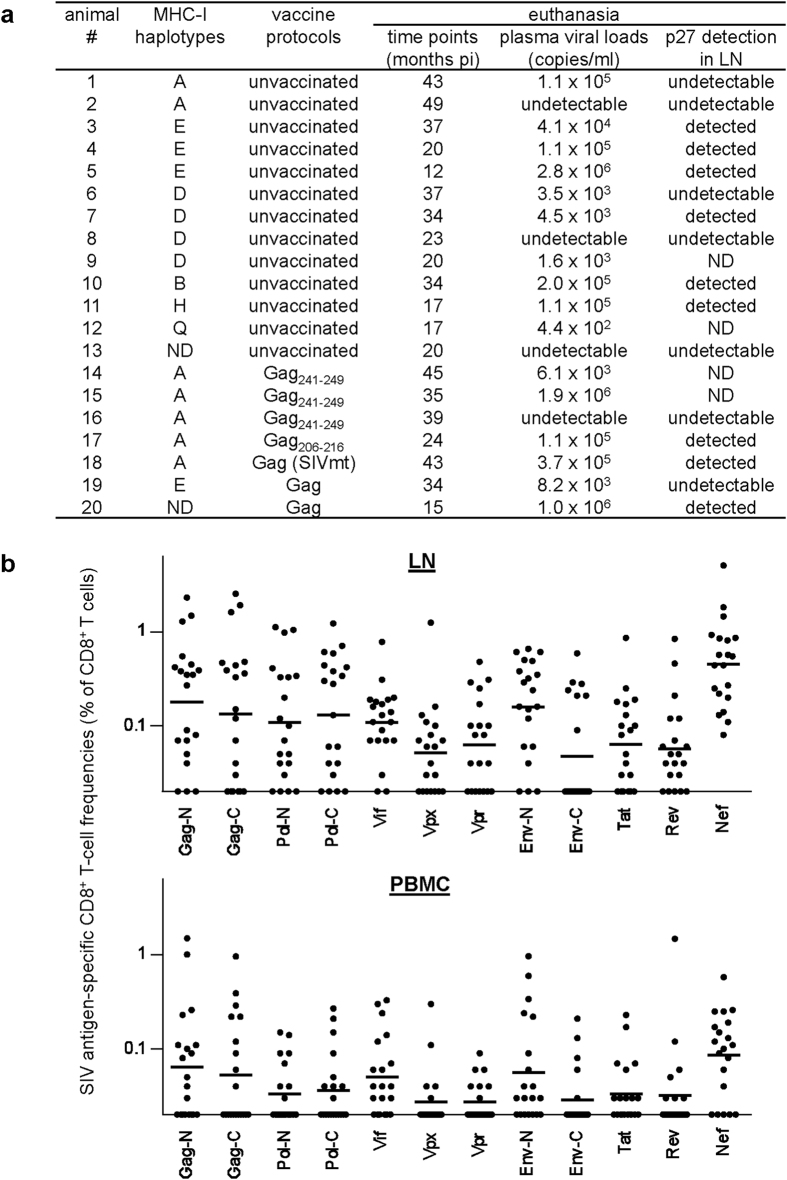
Macaques used in this study and SIV antigen-specific CD8^+^ T-cell responses in the chronic phase of SIV infection. (**a**) List of twenty macaques used in this study. Determined MHC-I haplotypes in individual animals are shown. Thirteen animals were unvaccinated. MHC-I haplotype A^+^ animals #14, #15, and #16 received a DNA-prime/SeV-boost vaccine eliciting Gag_241–249_-specific CD8^+^ T-cell responses. A^+^ animal #17 received a DNA-prime/SeV-boost eliciting Gag_206–216_-specific CD8^+^ T-cell responses. Animals #18, #19, and #20 received a DNA-prime/SeV-Gag-boost. Animal #18 was challenged with an SIV carrying five gag mutations^20^, while all other animals were infected with the wild-type SIVmac239. Plasma viral loads at euthanasia (at indicated months post-infection [pi]) are shown. Viral loads in macaques #4, #5, and #11 were previously described^38^,^41^. Results on immunohistochemistry analysis to detect SIV p27 antigen in the inguinal LNs obtained at euthanasia are also shown. ND, not determined. (**b**) SIV individual antigen-specific CD8^+^ T-cell responses in the twenty macaques at euthanasia. Frequencies of CD8^+^ T cells (% of CD8^+^ T cells) targeting Gag-N, Gag-C, Pol-N, Pol-C, Vif, Vpx, Vpr, Env-N, Env-C, Tat, Rev, and Nef, respectively in the inguinal LNs (upper panel) and PBMCs (lower panel) are shown.

We examined SIV antigen-specific CD8^+^ T-cell responses in the inguinal LNs and PBMCs obtained from twenty rhesus macaques in the chronic phase of SIV infection (Fig. 1). We used animals with varieties of MHC-I haplotypes because MHC-I genotypes are associated with target antigens for CD8^+^ T cells. The twenty macaques were consisting of thirteen unvaccinated including both non-controllers and controllers and seven vaccinated also including both non-controllers and controllers.

All the macaques showed detectable SIV Nef antigen-specific CD8^+^ T-cell responses in the LNs. CD8^+^ T-cell responses targeting the N-terminal half of SIV Gag (Gag-N), SIV Vif and the N-terminal half of SIV Env (Env-N) antigens were detected in seventeen or eighteen animals, whereas responses specific for other SIV antigens were detectable in less animals.

**Figure 2:**
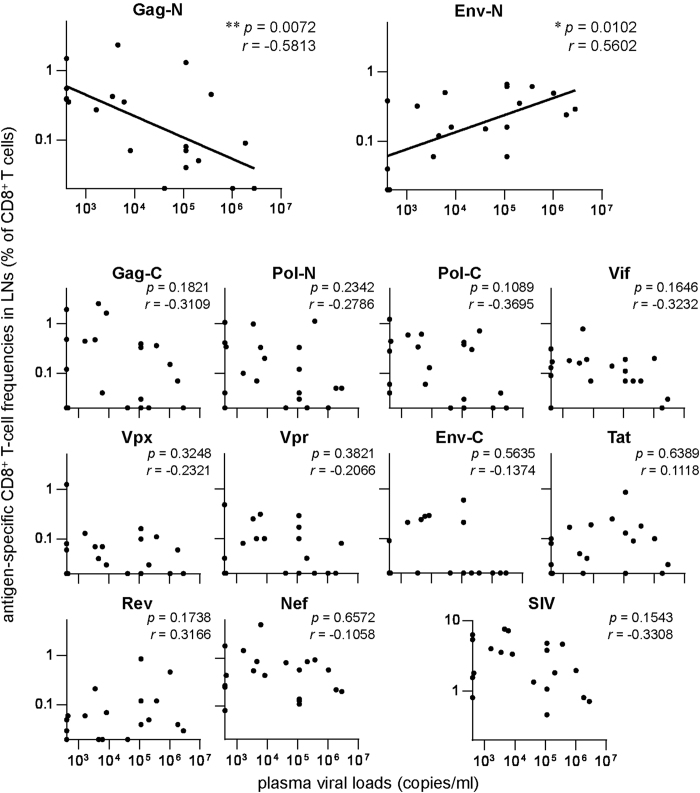
Correlation analyses between plasma viral loads and SIV antigen-specific CD8^+^ T-cell frequencies in LNs. Correlation was analyzed between viral loads and frequencies of CD8^+^ T cells targeting Gag-N, Gag-C, Pol-N, Pol-C, Vif, Vpx, Vpr, Env-N, Env-C, Tat, Rev, Nef, or whole SIV antigens in the inguinal LNs in the chronic phase of SIV infection. Viral loads were inversely correlated with Gag-N-specific CD8^+^ T-cell frequencies (*p* = 0.0072, *r* = −0.5813 by Spearman’s test) and positively correlated with Env-N-specific CD8^+^ T-cell frequencies (*p* = 0.0102, *r* = 0.5602), as shown in the top two panels.

Correlation analyses (Fig. 2) showed no association between plasma viral loads and whole SIV antigen-specific CD8^+^ T-cell frequencies (the sum of Gag-N-, Gag-C-, Pol-N-, Pol-C-, Vif-, Vpx-, Vpr-, Env-N-, Env-C-, Tat-, Rev-, and Nef-specific CD8^+^ T-cell frequencies) in the LNs in the chronic phase. However, we found an inverse correlation between viral loads and Gag-N-specific CD8^+^ T-cell frequencies (*p* = 0.0072, *r* = −0.5813 by Spearman’s test). In contrast, Env-N-specific CD8^+^ T-cell frequencies were positively correlated with viral loads (*p* = 0.0102, *r* = 0.5602). No significant correlation was observed between viral loads and frequencies of CD8^+^ T cells targeting other SIV antigens in LNs.

**Figure 3:**
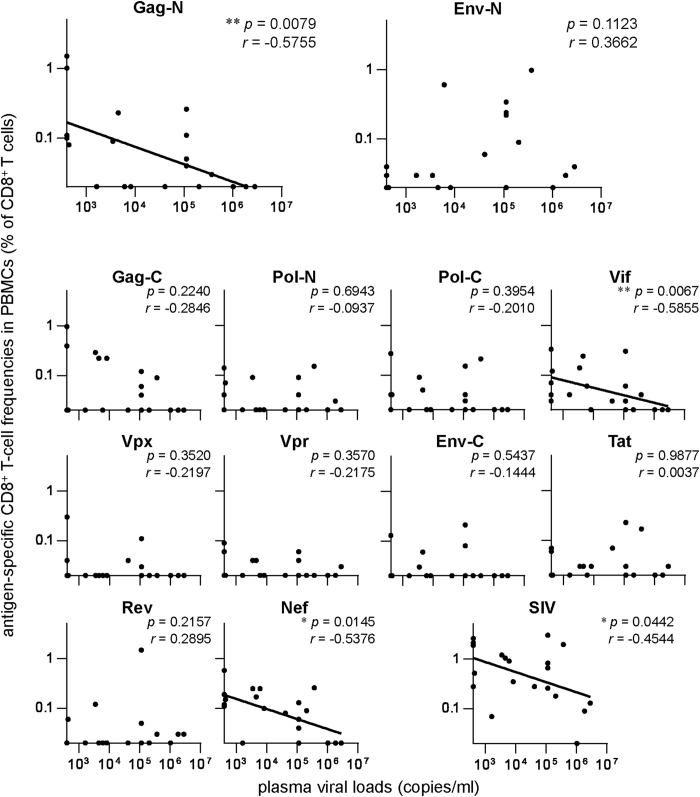
Correlation analyses between plasma viral loads and SIV antigen-specific CD8^+^ T-cell frequencies in PBMCs. Correlation was analyzed between viral loads and frequencies of CD8^+^ T cells targeting Gag-N, Gag-C, Pol-N, Pol-C, Vif, Vpx, Vpr, Env-N, Env-C, Tat, Rev, Nef, or whole SIV antigens in PBMCs in the chronic phase of SIV infection. Viral loads were inversely correlated with Gag-N-, Vif-, and Nef-specific CD8^+^ T-cell frequencies, respectively (*p* = 0.0079, *r* = −0.5755 on Gag-N; *p* = 0.0067, *r* = −0.5855 on Vif; *p* = 0.0145, *r* = −0.5376 on Nef by Spearman’s test). A marginal inverse correlation was also observed between viral loads and whole SIV antigen-specific CD8^+^ T-cell frequencies (*p* = 0.0442, *r* = −0.4544). No significant correlation was observed between viral loads and Env-N-specific CD8^+^ T-cell frequencies.

We also examined SIV antigen-specific CD8^+^ T-cell responses in peripheral blood mononuclear cells (PBMCs) of these animals (Fig. 3). We confirmed an inverse correlation between viral loads and Gag-N-specific CD8^+^ T-cell frequencies (*p* = 0.0079, *r* = −0.5755), but no significant correlation was observed between viral loads and Env-N-specific CD8^+^ T-cell frequencies in PBMCs. In addition, viral loads were inversely correlated with Vif- and Nef-specific CD8^+^ T-cell frequencies in PBMCs, respectively (Vif: *p* = 0.0067, *r* = −0.5855; Nef: *p* = 0.0145, *r* = −0.5376). An inverse correlation was also observed between viral loads and whole SIV antigen-specific CD8^+^ T-cell frequencies (*p* = 0.0442, *r* = −0.4544), which may reflect above inverse correlations between viral loads and Gag-N/Vif/Nef-specific CD8^+^ T-cell frequencies in PBMCs.

### Central memory and effector memory subsets of SIV antigen-specific CD8^+^ T cells in LNs

To see the basis of above observations, we investigated frequencies of CD28^+^ CD95^+^ (central memory) and CD28^−^CD95^+^ (effector memory) subsets of SIV antigen-specific CD8^+^ T cells in the LNs (Fig. S1a, which shows a representative gating schema for flow cytometric analysis). In LNs, the majority of SIV antigen-specific CD8^+^ T cells were CD28^+^ CD95^+^. However, CD28^−^CD95^+^ subsets in Env-N-specific CD8^+^ T cells were frequently observed. A positive correlation between viral loads and CD28^−^CD95^+^ subset frequencies in whole SIV antigen-specific CD8^+^ T cells was observed (*p* = 0.0449, *r* = 0.4530 by Spearman’s test) (Fig. S1b). We also examined frequencies of CD28^+^ CD95^+^ and CD28^−^CD95^+^ subsets of SIV antigen-specific CD8^+^ T cells in PBMCs, but no significant correlation was observed between viral loads and CD28^−^CD95^+^ subset frequencies in whole SIV antigen-specific CD8^+^ T cells in PBMCs (Fig. S1b).

**Figure 4:**
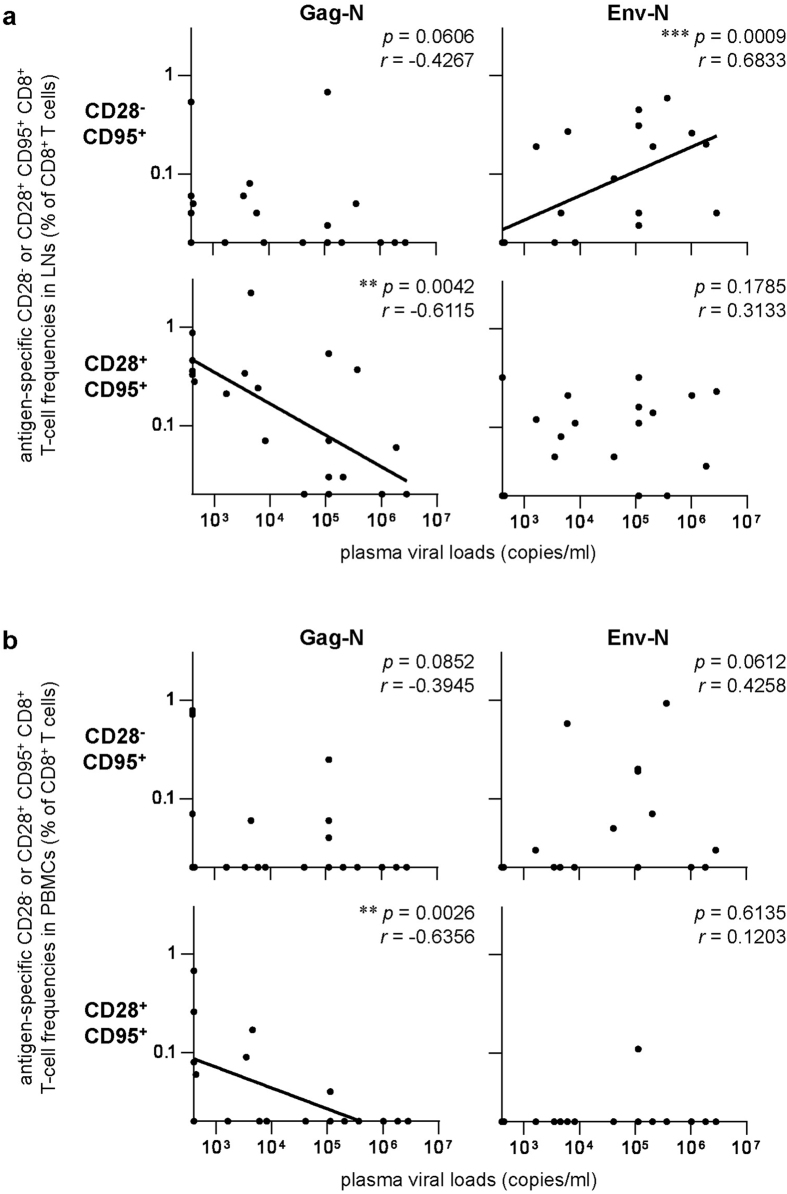
Correlation analyses between plasma viral loads and Gag-N- or Env-N-specific CD28^−^ or CD28^+^ CD95^+^ CD8^+^ T^−^cell frequencies. (**a**) Correlation analyses between viral loads and Gag-N- or Env-N-specific CD28^−^ or CD28^+^ CD95^+^ CD8^+^ T-cell frequencies in the inguinal LNs. Viral loads were inversely correlated with Gag-N-specific CD28^+^ CD95^+^ CD8^+^ T-cell frequencies (*p* = 0.0042, *r* = −0.6115 by Spearman’s test) and positively correlated with Env-N-specific CD28^−^CD95^+^ CD8^+^ T-cell frequencies (*p* = 0.0009, *r* = 0.6833). (**b**) Correlation analyses between viral loads and Gag-N- or Env-N-specific CD28^−^ or CD28^+^ CD95^+^ CD8^+^ T-cell frequencies in PBMCs. Viral loads were inversely correlated with Gag-N-specific CD28^+^ CD95^+^ CD8^+^ T-cell frequencies (*p* = 0.0026, *r* = −0.6356 by Spearman’s test). No significant correlation was observed between viral loads and Env-N-specific CD28^−^CD95^+^ CD8^+^ T-cell frequencies.

Correlation analyses showed an inverse correlation between plasma viral loads and Gag-N-specific CD28^+^ CD95^+^ (central memory) CD8^+^ T-cell frequencies in the LNs (*p* = 0.0042, *r* = −0.6115) (Fig. 4a). No significant correlation was observed between viral loads and Gag-N-specific CD28^−^CD95^+^ CD8^+^ T-cell frequencies. In contrast, we found a strong positive correlation between viral loads and Env-N-specific CD28^−^CD95^+^ (effector memory) CD8^+^ T-cell frequencies in the LNs (*p* = 0.0009, *r* = 0.6833), while no correlation was observed between viral loads and Env-N-specific CD28^+^ CD95^+^ CD8^+^ T-cell frequencies (Fig. 4a). In addition, viral loads were inversely correlated with Vif-specific CD28^+^ CD95^+^ CD8^+^ T-cell frequencies (*p* = 0.0257, *r* = −0.4972) (Fig. S2). No significant correlation was observed between viral loads and frequencies of CD28^−^CD95^+^ or CD28^+^ CD95^+^ CD8^+^ T cells targeting other SIV antigens.

Viral loads were also inversely correlated with Gag-N-specific CD28^+^ CD95^+^ CD8^+^ T-cell frequencies in PBMCs (*p* = 0.0026, *r* = −0.6356) (Fig. 4b). Remarkably, no correlation was observed between viral loads and Env-N-specific CD28^−^CD95^+^ or CD28^+^ CD95^+^ CD8^+^ T-cell frequencies in PBMCs (Fig. 4b). Furthermore, viral loads were inversely correlated with Vif- and Nef-specific CD28^+^ CD95^+^ CD8^+^ T-cell frequencies in PBMCs, respectively (Vif: *p* = 0.0315, *r* = −0.4818; Nef: *p* = 0.0343, *r* = −0.4750) (Fig. S3). An inverse correlation was also observed between viral loads and whole SIV antigen-specific CD28^+^ CD95^+^ CD8^+^ T-cell frequencies (*p* = 0.0097, *r* = −0.5632).

Above analyses were performed by using twenty SIV-infected animals consisting of thirteen unvaccinated and seven vaccinated. We then examined whether above results can be confirmed by using only the former unvaccinated animals. In the analyses using thirteen unvaccinated animals, plasma viral loads were inversely correlated with Gag-N-specific CD28^+^ CD95^+^ (central memory) CD8^+^ T-cell frequencies (*p* = 0.0329, *r* = −0.5810) and positively correlated with Env-N-specific CD28^−^CD95^+^ (effector memory) CD8^+^ T-cell frequencies in the LNs (*p* = 0.0112, *r* = 0.6964) (Fig. S4). No significant correlation was observed between viral loads and frequencies of Gag-N-specific CD28^−^CD95^+^ CD8^+^ T cells or Env-N-specific CD28^+^ CD95^+^ CD8^+^ T cells. These are consistent with the results obtained from the twenty macaques that are shown in Fig. 4a.

**Figure 5:**
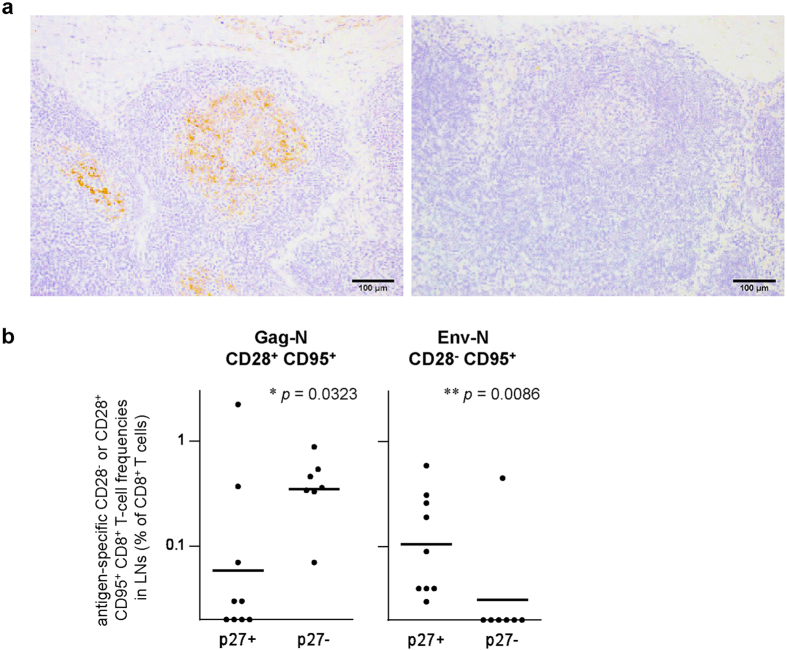
Comparison of Gag-N- or Env-N-specific CD8^+^ T-cell frequencies between p27-positive and p27-negative LNs. (**a**) Immunostaining by anti-p27 antibody. Representative p27-positive (left panel) and p27-negative (right panel) results are shown. In the left panel, p27 was detected in the germinal center. (**b**) Comparisons of Gag-N-specific CD28^+^ CD95^+^ CD8^+^ T-cell frequencies (left panel) and Env-N-specific CD28^−^CD95^+^ CD8^+^ T-cell frequencies (right panel) in p27-positive (n = 9) and p27-negative (n = 7) LNs. LNs with detectable p27 had significantly lower Gag-N-specific CD28^+^ CD95^+^ CD8^+^ T-cell (*p* = 0.0323 by Mann-Whitney U-test) and significantly higher Env-N-specific CD28^−^CD95^+^ CD8^+^ T-cell frequencies (*p* = 0.0086) than those without detectable p27.

Finally, we examined SIV Gag capsid p27 antigen in the LNs derived from sixteen animals by immunostaining. In nine of them, the p27 was clearly detected in the germinal center but not in the paracortical area (Fig. 5a). The antigen was detectable in most of the animals with more than 1.0 × 10^4^ copies/ml of plasma viral loads except for animal #1, but undetectable in those with less than 1.0 × 10^4^ copies/ml except for animal #7 (Fig. 1a). Comparison of T-cell responses showed significantly higher Gag-N-specific CD28^+^ CD95^+^ CD8^+^ T-cell frequencies in the LNs without detectable p27 than those with detectable p27 (*p* = 0.0323 by Mann-Whitney U-test) (Fig. 5b). In contrast, Env-N-specific CD28^−^CD95^+^ CD8^+^ T-cell frequencies were significantly higher in the LNs with detectable p27 (*p* = 0.0086) (Fig. 5b). Indeed, Gag-N-specific CD28^+^ CD95^+^ CD8^+^ T cells were positive in all the LNs without detectable p27, whereas Env-N-specific CD28^−^CD95^+^ CD8^+^ T cells were positive in all the p27-positive LNs but negative in six of the seven p27-negative LNs.

## Discussion

Cumulative studies have indicated strong suppressive pressure on HIV-1 replication by Gag-specific CD8^+^ T cells^9^,^27^,^28^,^29^,^30^. An inverse correlation between plasma viral loads and Gag-specific CD8^+^ T-cell responses in PBMCs was previously shown by a large cohort of HIV-1-infected individuals^22^,^23^,^24^. The present study found that viral loads were inversely correlated with Gag-N-specific CD8^+^ T-cell frequencies even in the inguinal LNs in a macaque AIDS model. Further analysis indicated that this is based on an inverse correlation between viral loads and Gag-N-specific central memory CD28^+^ CD95^+^ CD8^+^ T-cell frequencies in LNs. These results strongly support a notion that CD8^+^ T cells targeting the N-terminal half of Gag antigen including most of the N-terminal domain of capsid (CA) (refs 31 and 32) exert strong suppressive pressure on SIV replication in LNs. CD8^+^ T-cell responses targeting more localized regions in Gag-N may be more strongly associated with lower viral loads. LNs without detectable p27 had significantly higher frequency Gag-N-specific CD28^+^ CD95^+^ CD8^+^ T cells than those with detectable p27. In the latter LNs with detectable p27, Gag-N-specific CD28^−^CD95^+^ CD8^+^ T cells, even if induced, might be consumed to fight against SIV-infected cells.

Analysis of LNs revealed a positive correlation between plasma viral loads and Env-N-specific CD8^+^ T-cell frequencies. Remarkably, frequencies of the Env-N-specific effector memory CD28^−^CD95^+^ subset in CD8^+^ T cells in LNs were correlated with viral loads. This correlation was found by analysis of LNs but not confirmed in PBMCs. LNs with detectable p27 had significantly higher frequency Env-N-specific CD28^−^CD95^+^ CD8^+^ T cells than those without detectable p27. Indeed, Env-N-specific CD28^−^CD95^+^ CD8^+^ T cells were positive in all the p27-positive LNs but negative in most of the p27-negative LNs. These results indicate that Env-N-specific CD28^−^CD95^+^ CD8^+^ T-cell responses reflect SIV replication in LNs. This suggests a possibility that SIV replication results in induction of Env-N-specific CD8^+^ T-cell effectors, which fail to exert effector function to suppress viral replication and remain in LNs.

The present study indicates that induction of Env-N-specific CD8^+^ T cells in LNs does not contribute to suppression of SIV replication. We found association of viral loads with CD8^+^ T cells targeting the N-terminal half of Env including most of Env surface protein (SU, gp120) but not with those targeting the C-terminal half of Env including Env transmembrane protein (TM, gp41). Anti-SIV antibodies frequently target the former Env-N region, resulting in its higher diversity^33^,^34^. Thus, a possible explanation is that high diversity of Env-N-coding regions in viral genome may result in inefficient recognition and killing of target SIV-infected cells by Env-N-specific CD8^+^ T cells.

Our immunostaining detected SIV p27 antigens in the germinal center of the inguinal LNs. SIV-infected CD4^+^ T cells in the germinal center might have less chance to encounter SIV-specific CD8^+^ T cells compared to those in the paracortical area^35^. Then, it can be speculated that SIV replication in the germinal center that produces soluble gp120 antigens, resulting in induction of Env-N-specific CD8^+^ T cells in the paracortical area. This may also explain the association of Env-N-specific CD8^+^ T-cell responses with SIV replication in LNs.

In summary, we found an inverse correlation between plasma viral loads and Gag-N-specific central memory CD28^+^ CD95^+^ CD8^+^ T-cell frequencies. Furthermore, analysis of LNs revealed a positive correlation between viral loads and Env-N-specific effector memory CD28^−^CD95^+^ CD8^+^ T-cell frequencies in the chronic phase of SIV infection. LNs with detectable p27 antigen had lower Gag-N-specific CD28^+^ CD95^+^ CD8^+^ T-cell and higher Env-N-specific CD28^−^CD95^+^ CD8^+^ T-cell frequencies than those without detectable p27. These results suggest that core and envelope antigen-specific CD8^+^ T cells exhibit different patterns of interactions with HIV/SIV-infected cells.

## Methods

### Animal experiments

We used twenty Burmese rhesus macaques (*Macaca mulatta*) chronically infected with SIV (Fig. 1a) for the present study. Seventeen macaques of the twenty had been used in our previous SIV challenge experiments^20^,^36^,^37^,^38^,^39^,^40^,^41^. Animals with varieties of MHC-I haplotypes including a protective MHC-I haplotype, *90-010-Id* (D) (ref. 39), were used in this study. The determination of macaque MHC-I haplotypes was based on the family study in combination with the reference strand-mediated conformation analysis of *Mamu-A* and *Mamu-B* genes and detection of major *Mamu-A* and *Mamu-B* alleles by cloning the reverse transcription (RT)-PCR products as described before^42^,^43^. Confirmed MHC-I alleles consisting of MHC-I haplotypes *90-120-Ia* (A), *90-120-Ib* (B), D, *90-010-Ie* (E), *90-030-Ih* (H), and *89-002-Iq* (Q) were described before^38^,^43^.

Of the twenty macaques, thirteen were unvaccinated and seven were vaccinated. Of the latter seven, three macaques possessing the MHC-I haplotype A, which is associated with dominant SIV Gag_206–216_- and Gag_241–249_-specific CD8^+^ T-cell responses^20^,^38^, received a DNA prime followed by an intranasal boost with a Sendai virus (SeV) vector eliciting Gag_241–249_-specific CD8^+^ T-cell responses and one A-positive macaque received a DNA-prime/SeV-boost vaccine eliciting Gag_206–216_-specific CD8^+^ T-cell responses as described before^36^,^37^. The remaining three received a DNA prime followed by a boost with an SeV vector expressing SIVmac239 Gag (SeV-Gag) (ref. 20,40). After vaccination, one DNA/SeV-Gag-vaccinated macaque was intravenously challenged with an SIV that carries five gag mutations resulting in a L-to-S substitution at the 216th amino acid in Gag, a D-to-E at the 244th, an I-to-L at the 247th, an A-to-V at the 312th, and an A-to-T at the 373rd (ref. 20), while all other animals were intravenously challenged with the wild-type SIVmac239.

Animal experiments were carried out in the Institute for Virus Research, Kyoto University (IVRKU) and National Institute of Biomedical Innovation (NIBP; currently renamed National Institutes of Biomedical Innovation, Health and Nutrition [NIBIOHN]) with the help of the Corporation for Production and Research of Laboratory Primates after approval by the Committee on the Ethics of Animal Experiments of IVRKU and NIBP under the guideline for animal experiments at IVRKU, NIBP, and National Institute of Infectious Diseases, which is in accordance with the Guidelines for Proper Conduct of Animal Experiments established by Science Council of Japan ( http://www.scj.go.jp/ja/info/kohyo/pdf/kohyo-20-k16-2e.pdf). Blood collection, vaccination, and SIV challenge were performed under ketamine anesthesia. Animals were euthanized at the end of experiments or at the endpoint determined by reduction in 10% loss of body weight, diarrhea, and general weakness. Animals with less than 200 cells/μl of peripheral CD4^+^ T-cell counts were not included in this study. At euthanasia, animals were deeply anesthetized with pentobarbital under ketamine anesthesia, and then, whole blood was collected from left ventricle. The inguinal LNs were obtained at autopsy just after the euthanasia.

### Analysis of antigen-specific CD8^+^ T-cell responses

We measured antigen-specific CD8^+^ T-cell responses by flow cytometric analysis detecting gamma interferon (IFN-γ) induction after specific stimulation as described previously^40^. PBMCs or lymphocytes derived from the inguinal LNs were cocultured with autologous herpesvirus papio-immortalized B-lymphoblastoid cell lines (B-LCLs) pulsed with peptide pools (at a final concentration of 1 to 2 μM for each peptide) using panels of overlapping peptides spanning the entire SIVmac239 Gag-N (the N-terminal half of Gag [1st–265th amino acids]), Gag-C (the C-terminal half of Gag [255th–510th]), Pol-N (the N-terminal half of Pol [1st–531st]), Pol-C (the C-terminal half of Pol [521st–1060th]), Vif, Vpx, Vpr, Env-N (the N-terminal half of Env [1st–447th]), Env-C (the C-terminal half of Env [437th–879th]), Tat, Rev, and Nef amino acid sequences, respectively. Intracellular IFN-γ staining was performed with a CytofixCytoperm kit (BD) and fluorescein isothiocyanate (FITC)-conjugated anti-human CD4 (M-T477, BD), peridinin chlorophyll protein (PerCP)-conjugated anti-human CD8 (SK1, BD), allophycocyanin-Cy7 (APC-Cy7)-conjugated anti-human CD3 (SP34-2, BD), allophycocyanin (APC)-conjugated anti-human CD28 (CD28.2, Biolegend), phycoerythrin-Cy7 (PE-Cy7)-conjugated anti-human CD95 (DX2, eBioscience), and phycoerythrin (PE)-conjugated anti-human IFN-γ (4S.B3, Biolegend) monoclonal antibodies. Specific T-cell frequencies were calculated by subtracting non-specific IFN-γ^+^ T-cell frequencies from those after antigen-specific stimulation. As negative controls, we examined Gag-N- and Gag-C-specific CD8^+^ T-cell responses in eleven naive Burmese rhesus macaques. The “M + 2 × SD” value (where M is the mean and SD is the standard deviation) of these negative controls was 0.02% (of CD8^+^ T cells). Then, specific CD8^+^ T-cell frequencies lower than 0.02% of CD8^+^ T cells were considered negative.

### Detection of viral antigen in the inguinal lymph nodes

Viral antigens (Gag capsid [CA] p27) in inguinal LNs were determined by immunohistochemistry using a rabbit anti-SIV Gag p27 polyclonal antibody (prepared by us) and an anti-rabbit immunoglobulin (EnVision/HRP, DAKO Cytomation) (ref. 44). The sections were counterstained with hematoxylin.

### Statistical analysis

All statistical analyses were performed using Prism software version 4.03 (GraphPad Software, Inc.). Comparison of antigen-specific CD8^+^ T-cell frequencies was performed by Mann-Whitney U-test (significance levels set at P < 0.05). Correlation analysis was performed by Spearman’s test (significance levels set at *P* < 0.05).

## Additional Information

**How to cite this article**: Ishii, H. *et al.* Association of lymph-node antigens with lower Gag-specific central-memory and higher Env-specific effector-memory CD8^+^ T-cell frequencies in a macaque AIDS model. *Sci. Rep.* **6**, 30153; doi: 10.1038/srep30153 (2016).

## Supplementary Material

Supplementary Information
