# Supplementary material for: Association of lymph-node antigens with lower Gag-specific central-memory and higher Env-specific effector-memory CD8+ T-cell frequencies in a macaque AIDS model
Source: Sci Rep. 2016 Jul 25;6:30153. doi: 10.1038/srep30153 (PMC4958968; doi:10.1038/srep30153)
Supplement: Supplementary Information [file srep30153-s1.pdf]

Association of lymph-node antigens with lower Gag-specific central-memory and higher Env-specific effector-memory CD8+ T-cell frequencies in a macaque AIDS model

Hiroshi Ishii[1], Saori Matsuoka[1], Takushi Nomura[1,2], Midori Nakamura[1], Teiichiro Shiino[1], Yuko Sato[3], Naoko Iwata-Yoshikawa[3], Hideki Hasegawa[3], Kazuta Mizuta[4], Hiromi Sakawaki[4], Tomoyuki Miura[4], Yoshio Koyanagi[4], Taeko K. Naruse[5], Akinori Kimura[5] and Tetsuro Matano[1, 6]*

[1]AIDS Research Center, National Institute of Infectious Diseases, Tokyo 162-8640, Japan
[2]Center for AIDS Research, Kumamoto University, Tokyo 162-8640, Japan
[3]Department of Pathology, National Institute of Infectious Diseases, Tokyo 162-8640, Japan
[4]Institute for Virus Research, Kyoto University, Kyoto 606-8507, Japan
[5]Department of Molecular Pathogenesis, Medical Research Institute, Tokyo Medical and Dental University, Tokyo 113-8510, Japan
[6]The Institute of Medical Science, The University of Tokyo, Tokyo 108-8639, Japan

***Corresponding author:** Tetsuro Matano, AIDS Research Center, National Institute of Infectious Diseases, 1-23-1 Toyama, Shinjuku-ku, Tokyo 162-8640, Japan. Phone: +81-3-4582-2811; Fax: +81-3-5285-1165; E-mail address: tmatano@nih.go.jp.

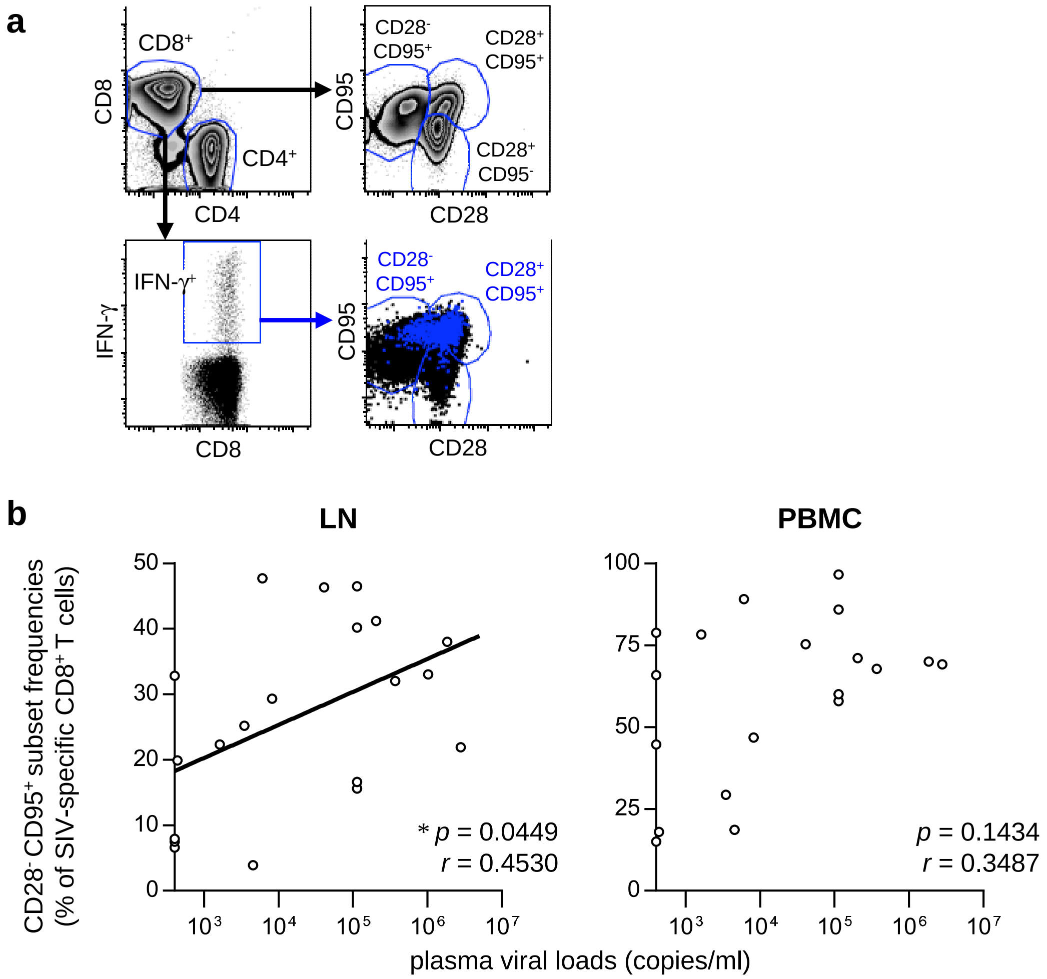

Supplemental Figure 1. CD28$^-$ CD95$^+$ subset frequencies in SIV antigen-specific CD8$^+$ T cells. (**a**) A representative gating schema for flow cytometric analysis of CD28$^-$ or CD28$^+$ CD95$^+$ subsets in SIV antigen-specific CD8$^+$ T cells. (**b**) Correlation analysis between plasma viral loads and CD28$^-$ CD95$^+$ subset frequencies in whole SIV antigen-specific CD8$^+$ T cells in the inguinal LNs (left panel) and PBMCs (right panel). Weak but significant correlation was observed in the LNs.

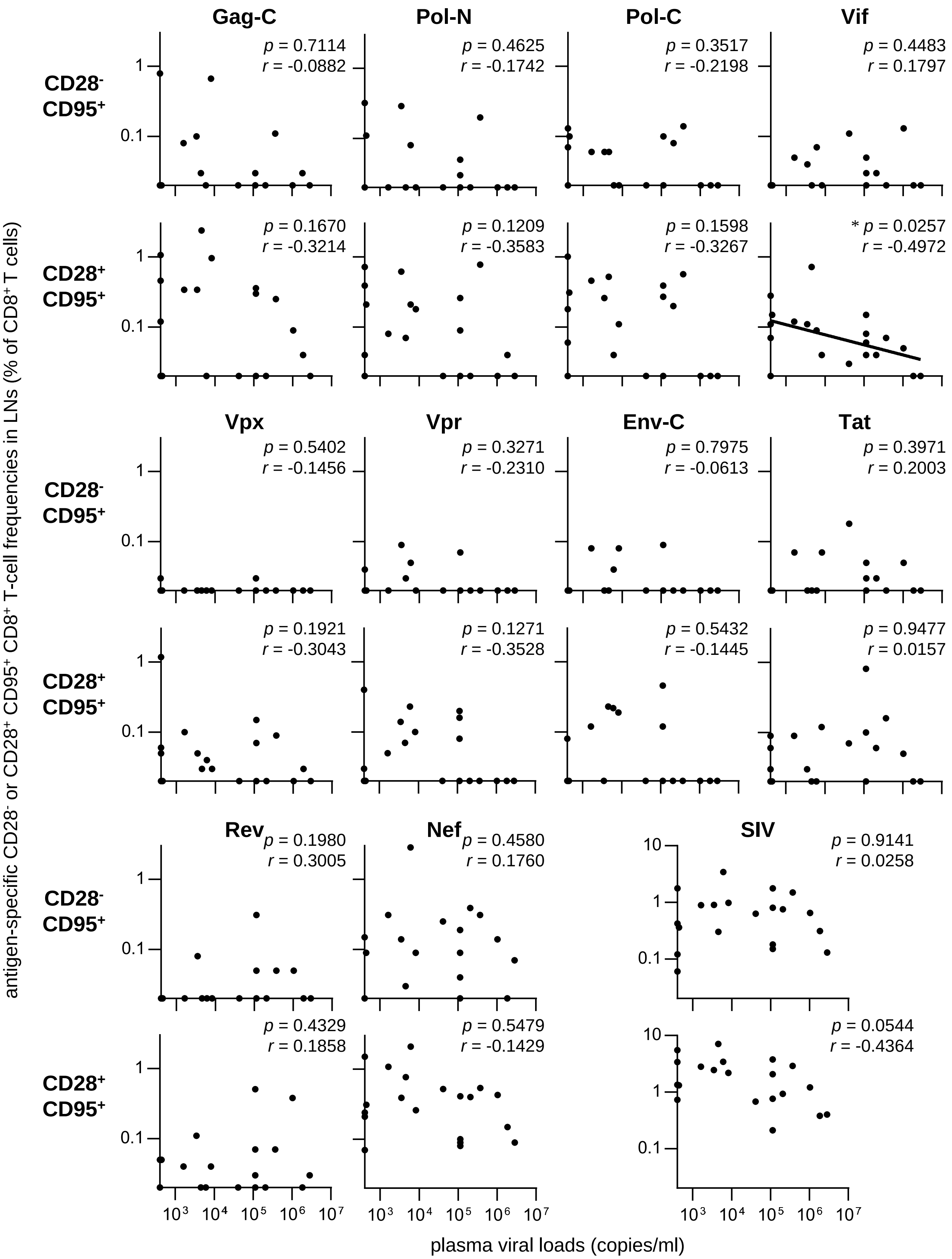

Supplemental Figure 2. Correlation analyses between plasma viral loads and antigen-specific CD28⁻ or CD28⁺ CD95⁺ CD8⁺ T-cell frequencies in the inguinal LNs.

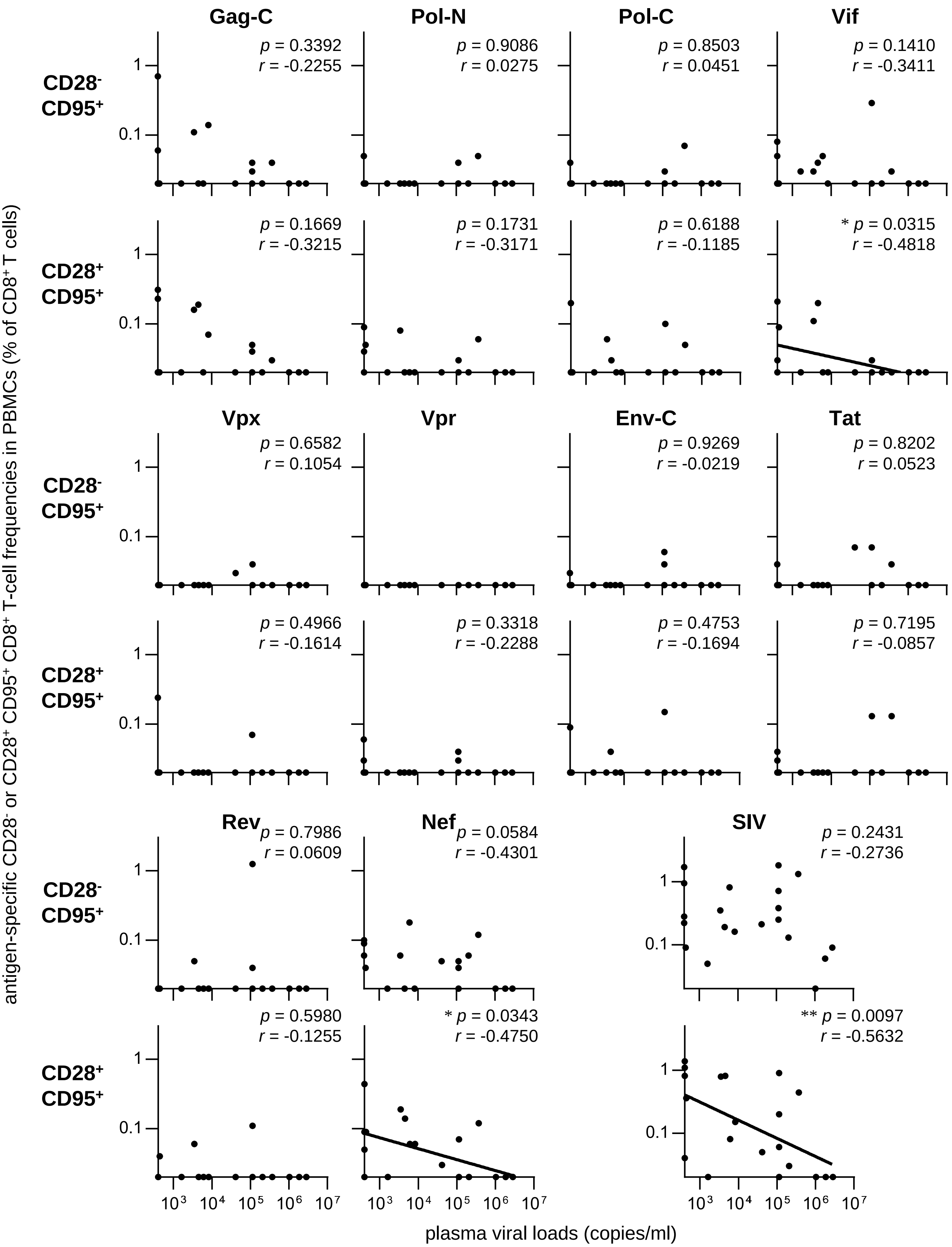

Supplemental Figure 3. Correlation analyses between plasma viral loads and antigen-specific CD28$^-$ or CD28$^+$ CD95$^+$ CD8$^+$ T-cell frequencies in PBMCs.

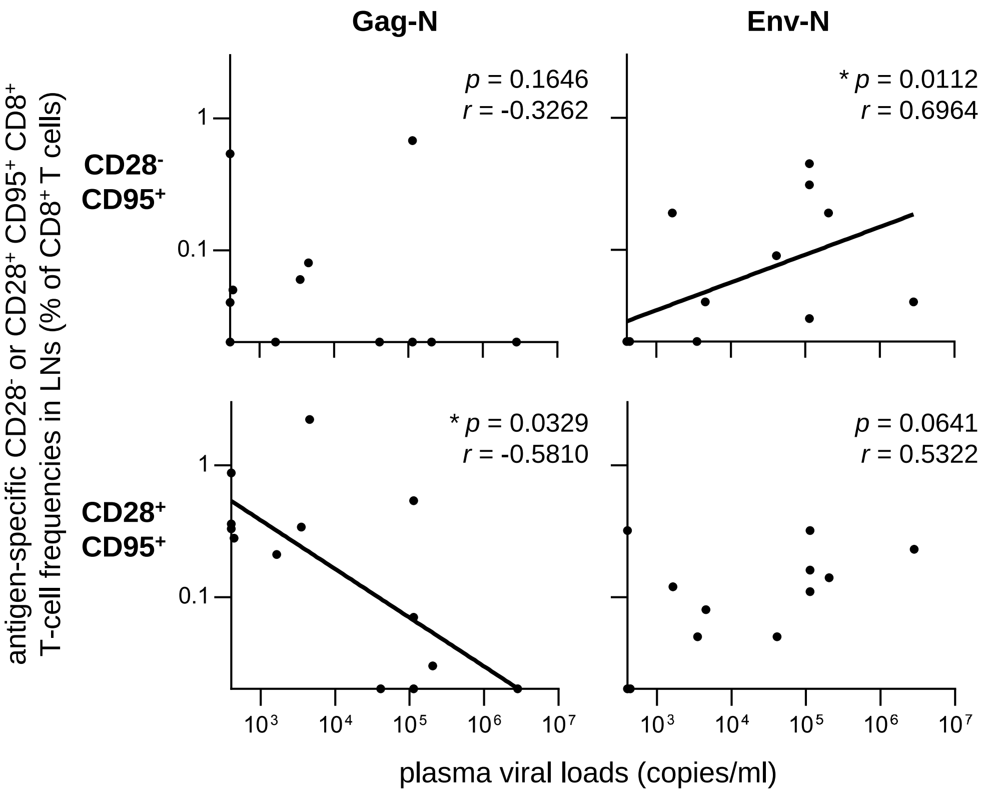

Supplemental Figure 4. Correlation analyses between plasma viral loads and Gag-N- or Env-N-specific $CD28^-$ or $CD28^+$ $CD95^+$ $CD8^+$ T-cell frequencies in the inguinal LNs in unvaccinated animals (n = 13). Viral loads were inversely correlated with Gag-N-specific $CD28^+$ $CD95^+$ $CD8^+$ T-cell frequencies and positively correlated with Env-N-specific $CD28^-$ $CD95^+$ $CD8^+$ T-cell frequencies.
